# Hepatocyte-specific angiotensinogen deficiency inhibits Western diet-induced liver steatosis with suppression of cell division in mice

**DOI:** 10.36922/gtm.6027

**Published:** 2025-04-10

**Authors:** Alex C. Pettey, Dien Ye, Sohei Ito, Alan Daugherty, Hong S. Lu, Hisashi Sawada

**Affiliations:** 1 Saha Cardiovascular Research Center, College of Medicine, University of Kentucky, Lexington, Kentucky, United States of America; 2 Saha Aortic Center, College of Medicine, University of Kentucky, Lexington, Kentucky, United States of America; 3 Department of Physiology, College of Medicine, University of Kentucky, Lexington, Kentucky, United States of America

**Keywords:** Angiotensinogen, Liver steatosis, Obesity, Transcriptomic analysis

## Abstract

Liver steatosis is a common cause of chronic liver disease. To investigate the molecular basis of hepatic steatosis, low-density lipoprotein receptor-deficient (LDLR −/−) mice were fed a Western diet (WD, 42% of calories from fat) for 5, 14, or 42 days and evaluated against mice fed a normal laboratory diet. Histological analyses revealed that steatosis was detected as early as 14 days of WD feeding. Bulk RNA sequencing demonstrated that WD feeding altered liver transcriptomes related to inflammation and cell adhesion consistent with the progression of liver steatosis. Previous studies determined that hepatocyte-specific deficiency of angiotensinogen (AGT), the unique substrate of the renin-angiotensin system (RAS), alleviates WD-induced hepatic steatosis in mice. However, the effects of hepatic AGT deficiency were not mimicked by pharmacological inhibition of the RAS, and the molecular mechanisms by which AGT deficiency protects against WD-induced steatosis is unknown. Therefore, liver transcriptomes were compared between hepatocyte-specific AGT-deficient mice (hepAGT −/−) and their wild-type littermates (hepAGT +/+) after 14 days of WD feeding. Gene ontology analyses showed that upregulated genes in hepAGT −/− mice were enriched for metabolic processes and downregulated genes were enriched for cell division pathways. The integration analysis of the two RNA sequencing data identified 5 key genes, *Smpd3*, *Dtl*, *Cdc6*, *Mki67*, and *Top2a*, which were primarily associated with cell division processes in hepAGT +/+ mice and were suppressed in hepAGT −/− mice. In conclusion, hepatic AGT deficiency downregulated genes related to cell division during the progression of liver steatosis.

## 1. Introduction

Liver steatosis, characterized by excess lipid accumulation within hepatocytes, is a common hepatic disease that is prevalent worldwide.^1–5^ Liver steatosis represents an independent risk factor for chronic liver diseases and many cardiovascular diseases.^6^ Multiple studies have shown that consumption of a diet rich in saturated fats has a significant positive correlation with steatosis in humans.^7,8^ Furthermore, there is compelling preclinical evidence that feeding with Western diet (WD), a laboratory diet enriched in saturated fats, induces liver steatosis in mice.^9^ Thus, WD feeding in combination with genetic mouse models provides a valuable approach to investigate specific molecular targets involved in the initiation and progression of liver steatosis.

Angiotensinogen (AGT) is the unique substrate of the renin-angiotensin system (RAS) and is derived predominantly from hepatocytes.^10–12^ AGT exerts a key role in WD-induced steatosis.^11,13^ Previous preclinical studies demonstrated that global and hepatocyte-specific AGT deficiency ameliorated WD-induced liver steatosis in both hypercholesteremic and normolipidemic mice.^11,14^ AGT hypomorphic mice had a drastic reduction of AGT plasma concentrations and were resistant to WD-induced increases in body weight, liver weight, and liver triglycerides.^11^ Hepatocyte-specific AGT deficiency decreased plasma AGT concentrations comparable to AGT hypomorphic mice and also conferred resistance to diet-induced body weight gain and liver steatosis.^11,14^ Of note, the effect of AGT deficiency on liver steatosis was not mimicked by inhibition of renin or the angiotensin II type 1 receptor (AT1R), which indicates that the effect is independent of angiotensin II stimulation of AT1R.^11,14,15^ Furthermore, in hepatocyte-specific AGT-deficient mice, restoration of plasma AGT concentrations by infection with an adeno-associated virus expressing des(AngI)AGT, a cleaved form of AGT that lacks the portion corresponding to angiotensin I (AngI), resulted in restoration of WD-induced body weight gain and liver steatosis comparable to expression of full-length AGT.^11^ Importantly, despite the clear contribution of hepatic AGT to development of WD-induced steatosis, few studies have investigated functions of AGT outside of the production of RAS peptides, and the mechanism by which hepatocyte-specific AGT deletion inhibits liver steatosis remains unclear. Therefore, it is critical to identify potential molecules driving WD-induced liver steatosis through AGT.

In the present study, to identify target pathways and molecules, we employed a two-armed bulk RNA sequencing approach in mice. First, the temporal alteration of hepatic transcriptomes in response to WD was evaluated at different intervals of WD feeding in a hypercholesterolemic mouse model. Second, hepatic transcriptomes of hypercholesterolemia were compared between mice with AGT deletion in hepatocytes and their wild-type littermates. Last, the two RNA sequencing datasets were integrated to determine key processes that were altered by hepatic AGT deficiency during initiation of WD-induced steatosis.

## 2. Materials and methods

### 2.1. Mice

AGT floxed mice, with and without a transgene expressing Cre under the control of the albumin promoter (Alb-Cre; hepAGT −/− and hepAGT +/+, respectively), on a low-density lipoprotein-receptor deficient (LDLR −/−, #002207, The Jackson Laboratory, USA) background were developed and bred as described previously.^11^ Since liver steatosis is often observed during childhood, with the mean age of diagnosis being 11 – 13 years old in pediatric cases, the present study used mice at 7 weeks of age, comparable to humans at approximately 13 years of age.^16–20^ Mice were maintained in a barrier facility on a light: dark cycle of 14:10 h (ambient temperature of 21°C) and fed a normal mouse laboratory diet (fat 6.2% wt/wt; Diet #2918; Inotiv, USA). Littermates were used for all experiments. Male mice were used based on our previous study that found no sex-specific differences in hepAGT mice.^11^ To induce hypercholesterolemia, mice at 7 weeks of age were fed a diet supplemented with saturated fat (milk fat 21% wt/wt) and cholesterol (0.2% wt/wt; Diet #TD.88137, Inotiv, termed “Western diet”) for 5, 14, or 42 days. Mice were euthanized using a ketamine/xylazine cocktail (90 mg/kg #11695–6840-1, 10 mg/kg #11695–4024-1, respectively; Covetrus, USA). To minimize transcriptomic alterations following euthanasia, cold saline (8 – 10 mL) was perfused from the left ventricle, and livers were harvested immediately.

### 2.2. Histological analyses

Liver portions from the left lobe were collected and processed for histological analysis. For hematoxylin and eosin (H&E) staining, samples were fixed in paraformaldehyde (4% w/v), incubated with ethanol (70% v/v) for 24 h, embedded in paraffin, and sectioned (5 μm). Sections were subsequently deparaffinized using limonene (#183164, Millipore Sigma, Germany) followed by two washes with ethanol (100% v/v). Sections were then stained with Eosin Y (#ES709, Azer Scientific) for 2 min and counterstained with Mayer’s hematoxylin for 10 s (#26043–06, Electron Microscopy Sciences). Coverslips were applied using Permount (#SP-15, Fisher Scientific, USA). For Oil Red O staining, liver samples were embedded in optimal cutting temperature compound (#4585, Fisher Scientific), frozen at −20°C, and sectioned (10 μm). Sections were fixed in neutral-buffered formalin (10% w/v) and dehydrated in isopropanol (60% v/v) for 5 min. Next, sections were stained with filtered Oil Red O (#O0625, Millipore Sigma; 0.15% w/v in 60% isopropanol) for 10 min, destained in isopropanol (60% v/v) for 2 min, and counterstained in Mayer’s hematoxylin (#26043–06, Electron Microscopy Sciences, USA) for 30 s. Coverslips were applied with warmed glycerol gelatin (#GG1, Millipore Sigma). Representative microscopic images were captured using an Eclipse NI microscope with a DS-Ri2 camera (Nikon, Japan). For quantification of Oil Red O, whole liver sections were imaged using a Z7 slide scanner (Zeiss, Germany). Oil Red O staining was quantified using an RGB color threshold (red: 0 – 255, green: 0 – 255, blue: 0 – 185) and normalized against total tissue area, defined by total pixels – white background pixels (red: 250 – 255, green: 250 – 255, blue: 250 – 255), using NIS-Elements AR (v4.51.00, Nikon).

### 2.3. Bulk RNA sequencing

Liver samples were snap-frozen in a homogenization solution (#Z305H, Promega, USA) containing 1-thioglycerol (#A208B, Promega). Subsequently, messenger RNA (mRNA) was extracted using Maxwell RSC simplyRNA Tissue Kits (#AS1340, Promega) according to the manufacturer’s protocol. Samples were processed individually for bulk RNA sequencing, with single mice representing independent experimental replicates. RNA samples were shipped to Novogene (USA) for bulk mRNA sequencing. The sequencing library was generated from total mRNA (1 μg) using NEBNext Ultra^™^ RNA Library Prep Kits for Illumina (New England BioLabs, USA). cDNA libraries were then sequenced by a Next Generation NovaSeq platform, (HWI-ST1276, Illumina, USA), in a pair-end fashion to reach more than 1,500,000 reads. FASTQ sequence data were mapped to the reference mouse genome using STAR (v2.6.1d; https://academic.oup.com/bioinformatics/article/29/1/15/272537?login=false) and quantified using FeatureCounts (v1.5.0-p3; https://academic.oup.com/bioinformatics/article/30/7/923/232889?login=false). Bulk RNA sequencing data (raw FASTQ and aligned data) are publicly available at the gene expression omnibus repository (GEO accession number: GSE291082).

### 2.4. Statistical analysis

Data are presented as median and 25^th^/75^th^ percentiles. Down triangles represent biological replicates. Statistical analyses of Oil Red O quantification and individual target differentially expressed genes (DEGs) were performed using SigmaPlot (v15.0, SYSTAT Software Inc., USA). Normality and homogeneity of variance were assessed by Shapiro–Wilk and Brown–Forsythe tests, respectively. Data with confirmed normal distribution and homogeneous variance were analyzed by one-way analysis of variance (ANOVA) followed by Holm–Sidak test. Data that failed tests for normality or homogeneity of variance were analyzed by Kruskal–Wallis one-way ANOVA on Ranks with Dunn’s method. *p*<0.05 was considered statistically significant.

Bulk RNA sequencing data were analyzed on R (v4.1.0).^21,22^ Ensembl gene identifiers with no detected read counts in any sample were excluded. Read count data were normalized using the TMM method in edgeR package (v3.36.0) to adjust for biases in library size and composition. P-values were calculated using either “glmQLFTest” for multi-group or “exactTest” for two-group comparisons in edgeR. Since the present study aimed to profile genes with a high potential for interaction with AGT, miscellaneous transcripts such as duplicated, unnamed, ribosomal, and mitochondrial genes were removed. Criteria for removal was based on searches within Ensembl gene identifiers for “NA,” duplicated identifiers, or identifiers containing: “Gm[0 – 9],” “[0 – 9]Rik,” “RP[2 – 9],” or “mt-.” Subsequently, *p*-values were adjusted using the false discovery rate (FDR) method. FDR-adjusted *p*<0.05 was considered statistically significant. Gene ontology (GO) enrichment analysis of biological processes was performed using the clusterProfiler package (v4.2.2) after mapping Entrez gene identifiers with the org.Mm.eg.db package (v3.14.0) on R (v4.1.0).^23–25^ Chord plot illustration was performed using the SRplot online tool.^26^

## 3. Results

### 3.1. Macrovesicular liver steatosis was present after 14 days of WD feeding in hepAGT +/+ mice

**Figure 1.**
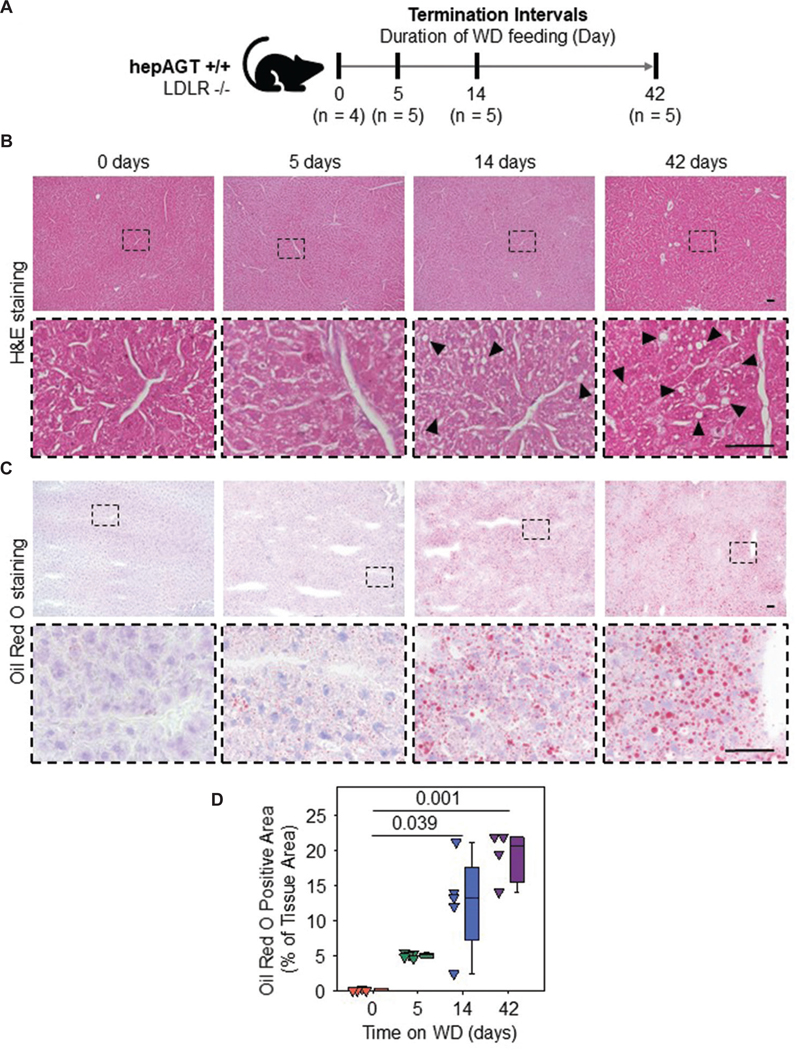
Macrovesicular liver steatosis was present after 14 days of WD feeding in hepAGT +/+ mice. (A) Albumin-Cre 0/0; angiotensinogen floxed (f/f) mice (hepAGT +/+) on an LDLR −/− background were fed WD for 0 (*n* = 4), 5 (*n* = 5), 14 (*n* = 5), or 42 (*n* = 5) days. (B) Representative images of H&E and (C) Oil Red O staining in livers. Black arrowheads indicate large intrahepatic vacuoles. Scale bars = 50 μm. (D) Quantification of Oil Red O staining-positive area. Numbers in the panel (D) are p-values calculated by Kruskal–Wallis test followed by Dunn’s method. Abbreviations: H&E: Hematoxylin and eosin; LDLR: Low-density lipoprotein receptor; ND: Normal laboratory diet; WD: Western diet.

We first determined the temporal evolution of WD-induced liver steatosis in LDLR −/− mice. WD-induced liver steatosis is closely associated with body weight gain,^27,28^ and our previous study revealed that WD-induced body weight gain begins after 14 days of WD feeding.^14^ We therefore harvested liver tissues from LDLR −/− mice at selected intervals of WD feeding, including baseline and day 14 (Figure 1A). H&E staining did not detect discernable pathologies in livers at baseline and 5 days of WD feeding. At 14 days of WD feeding, intracellular vacuoles were detectable in a substantial portion of hepatocytes, indicating macrovesicular steatosis. At 42 days of WD feeding, intrahepatic vacuoles of increased size were present abundantly (Figure 1B). Oil Red O staining was then performed to assess neutral lipid accumulation in livers. Neutral lipids were minimally detected in baseline livers. At 5 days of WD feeding, punctate lipid droplets were diffusely present within hepatocytes without evident displacement of nuclei. However, we did not detect a statistically significant difference in the extent of Oil Red O-positive area between baseline and 5 days of WD feeding (Figure 1C and D). At 14 days of WD feeding, a subset of hepatocytes displayed large neutral lipid droplets surrounded by apparently displaced nuclei, indicative of macrovesicular steatosis. At 42 days of WD feeding, macrovesicular steatosis was apparent in most hepatocytes (Figure 1C). Oil Red O-positive areas were increased significantly at 14 and 42 days of WD feeding compared to baseline (Figure 1D). These data suggest that 5, 14, and 42 days of WD feeding induce liver steatosis corresponding to pre-pathological, initiation, and advanced phases, respectively, in hypercholesterolemic mice.

### 3.2. WD altered the liver transcriptome related to inflammation consistent with the progression of steatosis

**Figure 2.**
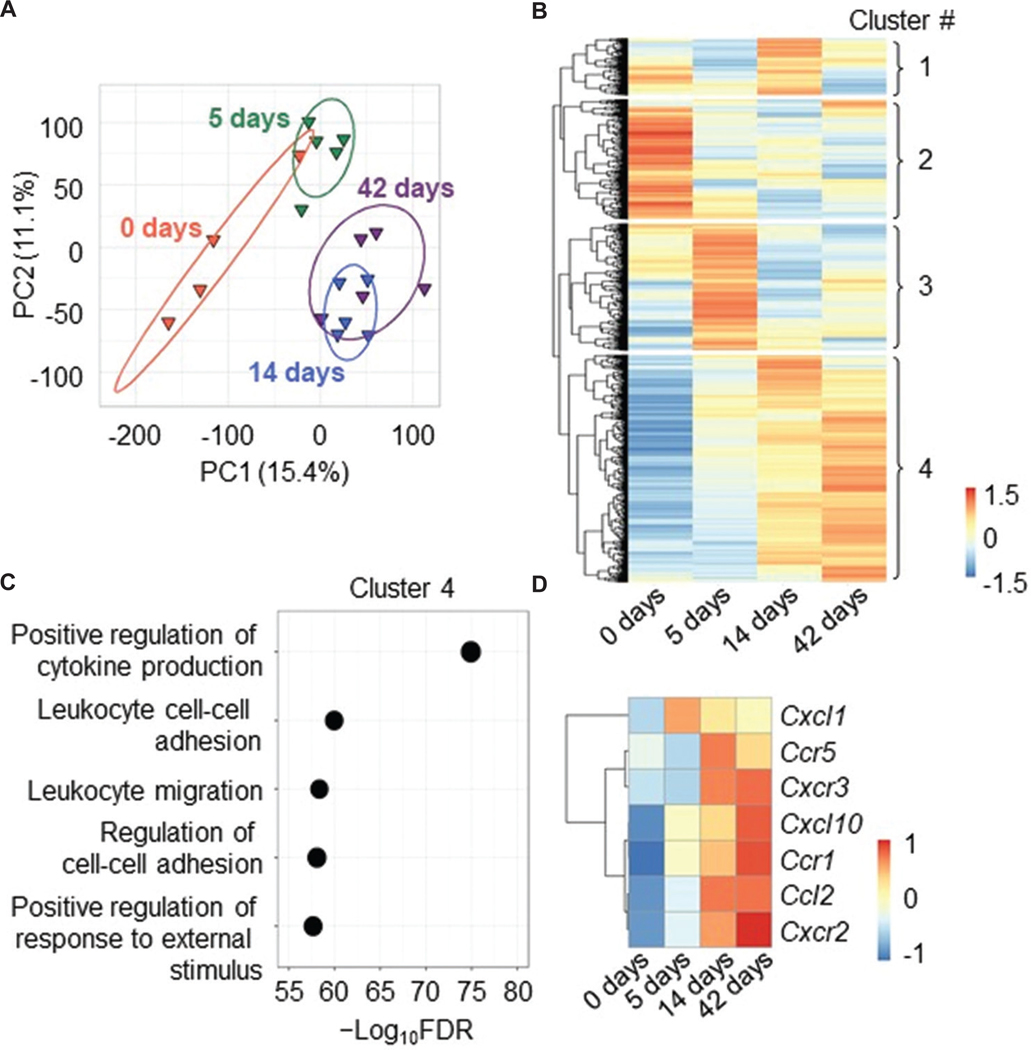
WD altered the liver transcriptome related to inflammation consistent with the progression of steatosis. (A) Principal component analysis of liver transcriptomes of hepAGT +/+ mice fed WD for 0, 5, 14, or 42 days. Numbers in parentheses indicate the percent of variation explained by the graphed PCs. (B) Z-scored heatmap of all DEGs. DEG cluster numbers were assigned by hierarchical clustering analysis. (C) Top five annotations in gene ontology analysis for biological processes using DEGs in Cluster 4. (D) Z-scored heatmap of key inflammatory cytokines and chemokines within detected DEGs. *n* = 4 – 5 mice/group. Abbreviations: DEG: Differentially expressed gene; FDR: False discovery rate; hepAGT +/+: Albumin-Cre 0/0 angiotensinogen floxed (f/f) low-density lipoprotein-receptor-deficient background; PC: Principal component; WD: Western diet.

**Figure A1.**
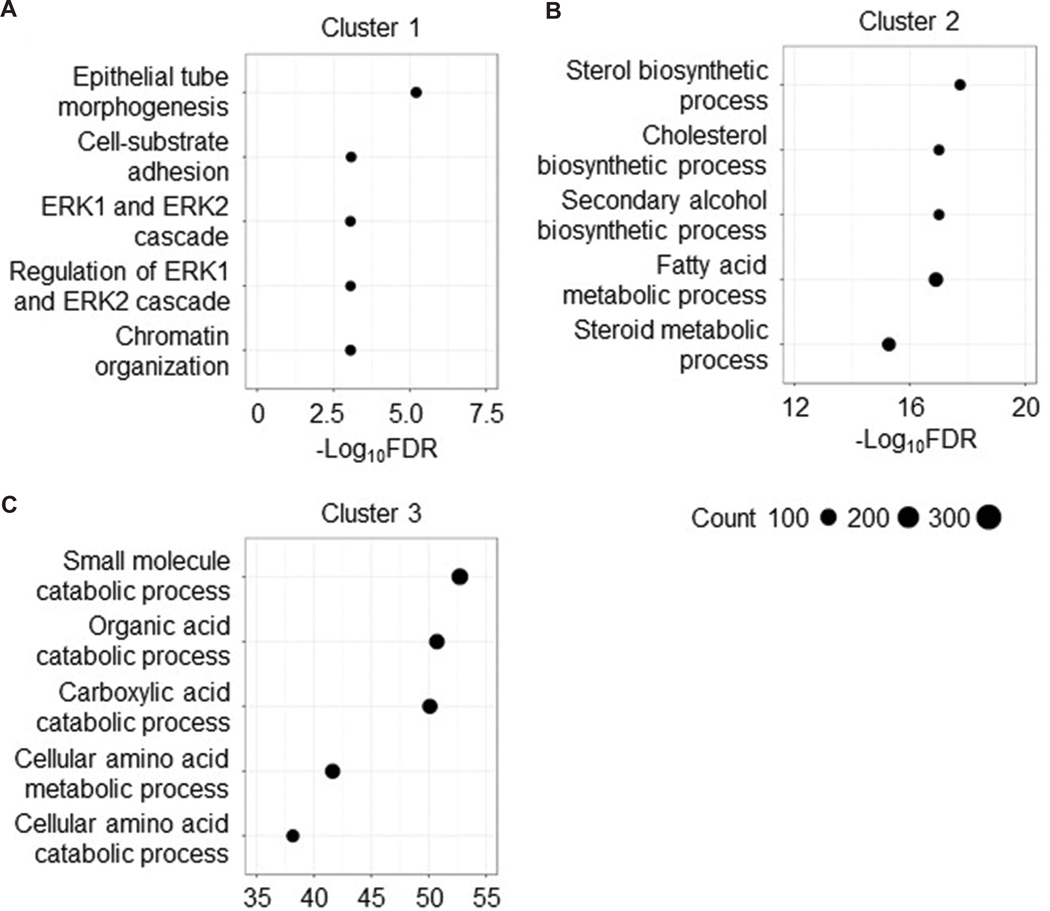
Gene ontology enrichment analysis for biological processes within subclustered DEGs of hepAGT +/+ mice fed WD for 5, 14, or 42 days and compared against mice fed for 0 days. DEG clusters were assigned by hierarchical clustering analysis. Top five annotations in gene ontology analysis for biological processes using DEGs in Cluster 1 (A), Cluster 2 (B), and Cluster 3 (C). *n* = 4 – 5 mice/group. Abbreviations: DEG: Differentially expressed gene; FDR: False discovery rate; hepAGT +/+: Albumin-Cre 0/0 angiotensinogen floxed (f/f) low-density lipoprotein-receptor-deficient background; WD: Western diet.

To identify key molecules driving WD-induced liver steatosis, bulk RNA-sequencing was performed in livers from LDLR −/− mice fed WD for 0, 5, 14, or 42 days. The bulk RNA sequencing identified 31,403 genes. Principal component analysis using unfiltered transcriptomes revealed distinct transcriptomic alterations in mice fed WD for 14 or 42 days compared to those fed WD for 5 days or baseline controls (Figure 2A), with the first two principal components explaining 26.5% of the variance among samples (15.4% + 11.1%). These data suggest that WD induces a transcriptomic shift within 14 days of feeding. Then, DEG analyses were performed among all four groups. There were 5,256 DEGs, and hierarchical clustering identified four distinct clusters in the DEGs (Figure 2B). Cluster 1 contained DEGs that were increased transiently by WD feeding at the initiation phase of liver steatosis. Meanwhile, DEGs in Cluster 2 were decreased by WD feeding. DEGs in Cluster 3 were increased transiently by WD feeding before the initiation phase of steatosis. Of note, Cluster 4 was composed of genes that were increased by WD feeding concurrent with steatosis development. GO enrichment analysis for biological processes was then performed to characterize the DEGs within each cluster. DEGs in Cluster 1 were associated mainly with epithelial tube morphogenesis and cell-substrate adhesion (Figure A1[A]). DEGs in Cluster 2 were related to sterol and secondary alcohol biosynthetic processes as well as fatty acid and steroid metabolic processes (Figure A1[B]). DEGs in Cluster 3 were related to catabolic processes (Figure A1[C]). Interestingly, in Cluster 4 which was consistent with the progression of liver steatosis, DEGs were related to inflammation and included major cytokines and chemokines, such as *Ccl2* and *Cxcl1* (Figure 2C and D).

### 3.3. Hepatocyte-specific AGT deficiency increased the hepatic transcriptome related to metabolic processes and suppressed cell division processes at 14 days of WD feeding

**Figure 3.**
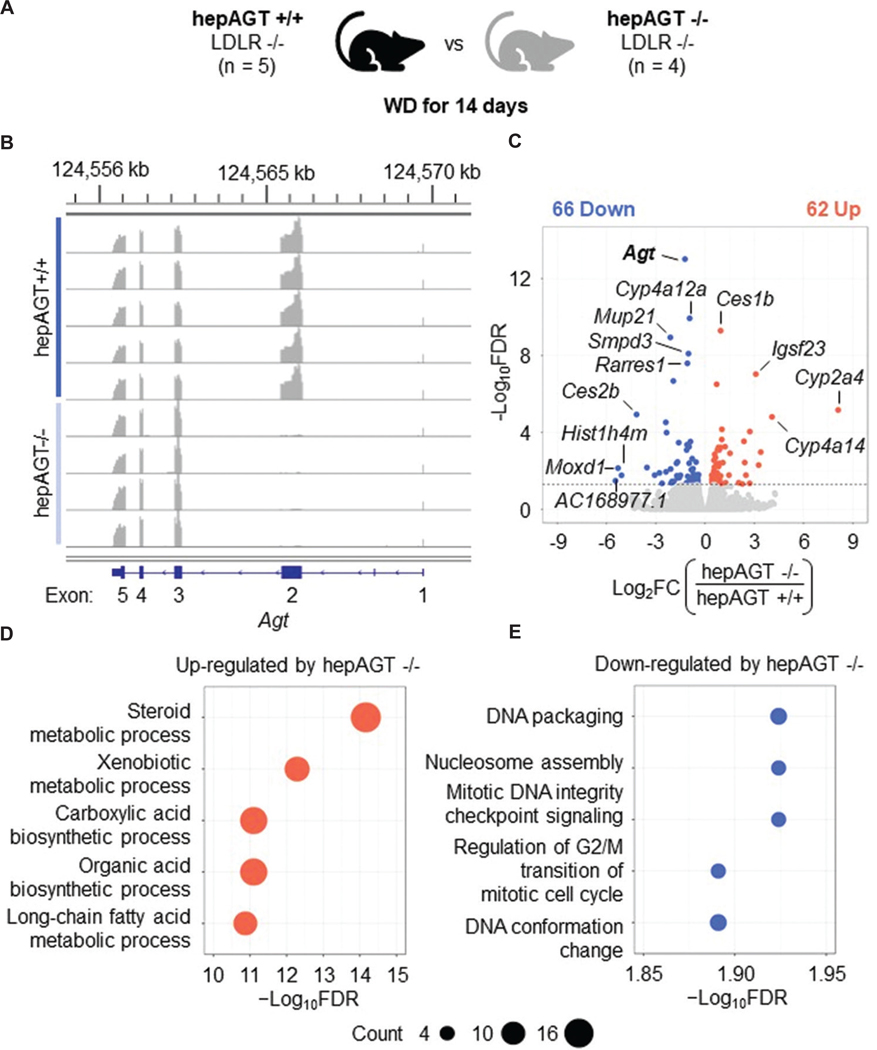
Hepatocyte-specific AGT deficiency increased the hepatic transcriptome related to metabolic processes and suppressed cell division processes at 14 days of WD feeding. (A) Livers of albumin-Cre 0/0; angiotensinogen floxed (f/f) mice (hepAGT +/+) and albumin-Cre 1/0; angiotensinogen floxed (f/f) mice (hepAGT −/−) on a low-density lipoprotein-receptor deficient (LDLR −/−) background fed WD for 14 days were analyzed by bulk RNA sequencing. (B) Visualization of read coverage on mouse *Agt* in hepAGT +/+ and −/− mice fed WD for 14 days. (C) Volcano plot of all DEGs between hepAGT +/+ versus −/− mice at 14 days of WD. Top 5 annotations in gene ontology analysis for biological processes within (D) upregulated and (E) downregulated DEGs. *n* = 4 – 5 mice/group. Abbreviations: AGT: Angiotensinogen; DEG: Differentially expressed gene; FC: Fold change; FDR: False discovery rate; WD: Western diet.

We next investigated the impact of hepAGT−/− on WD-induced transcriptomic alterations in liver. H&E and Oil Red O staining revealed that WD-induced liver steatosis becomes discernible after 14 days of WD feeding (Figure 1B-D). Therefore, to determine molecular mechanisms by which hepatocyte-derived AGT contributes to disease initiation, additional bulk RNA sequencing was performed using livers from hepAGT −/− mice after 14 days of WD feeding. DEG analyses were conducted by comparing these newly generated data with the previously sequenced transcriptomes of hepAGT +/+ mice after the same interval of 14 days of WD feeding (Figure 3A). *Agt* mRNA deletion was verified by reduction of mRNA reads aligned to exon 2 of *Agt* in hepAGT −/− mice (Figure 3B). Despite the significant reduction, only 128 DEGs were detected between genotypes. Sixty-two DEGs were upregulated and 66 DEGs were downregulated in hepAGT −/− mice compared to wild-type (Figure 3C). GO enrichment analysis revealed that genes upregulated in hepAGT −/− mice were related to metabolic and biosynthetic processes (Figure 3D). Meanwhile, downregulated genes were related to cell division processes (Figure 3E).

### 3.4. Cell division-related genes elevated by WD feeding were suppressed in hepatocyte-specific AGT-deficient mice at 14 days of WD

**Figure 4.**
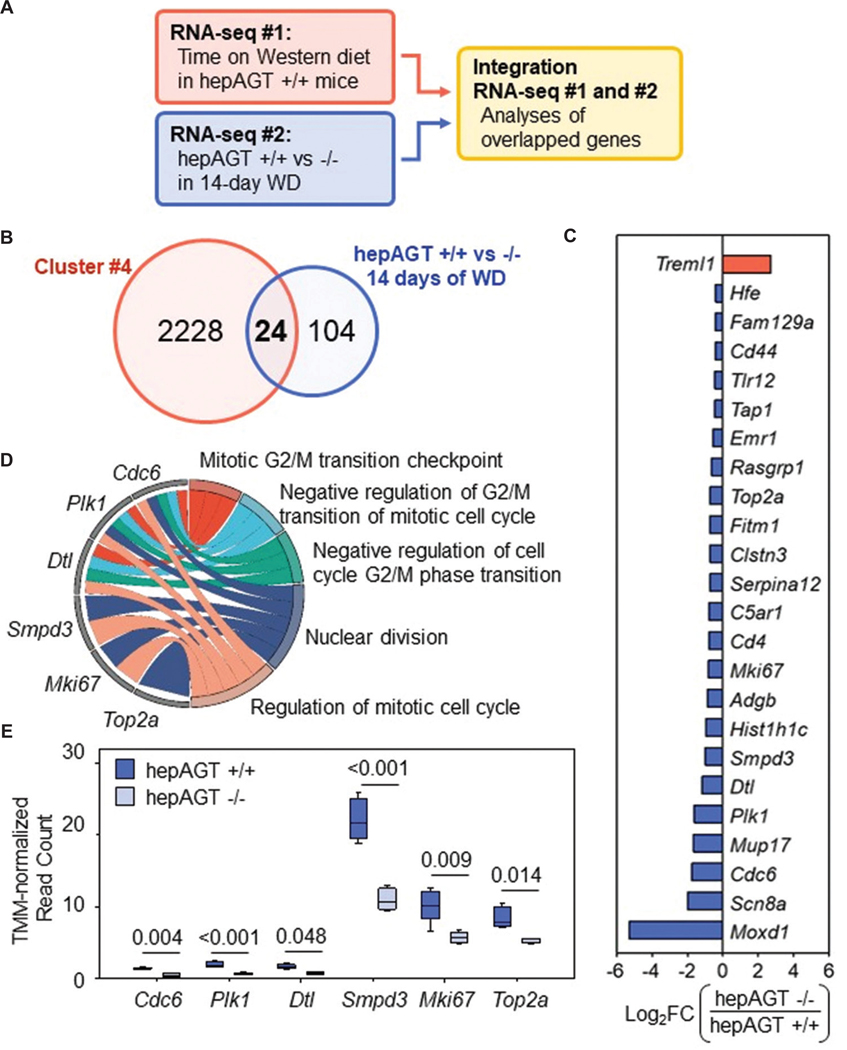
Cell division-related genes elevated by WD feeding were suppressed in hepatocyte-specific AGT-deficient mice at 14 days of WD. (A) Flowchart of two-armed bulk RNA sequencing. WD-induced liver transcriptomic alterations were analyzed in hepAGT +/+ mice after 5, 14, and 42 days of WD feeding compared against 0 days of WD. Liver transcriptomic alterations due to hepatic AGT deficiency were analyzed after 14 days of WD feeding by comparing hepAGT −/− mice against hepAGT +/+ mice. Both RNA sequencing data were integrated to identify target genes. (B) Venn diagram for overlapped DEGs from combined transcriptomic analyses. Cluster 4 represents DEGs with upregulation consistent with steatotic progression in hepAGT +/+ mice. hepAGT −/− versus +/+ represents DEGs altered in hepAGT −/− mice at 14 days of WD. (C) Log_2_FC alteration in hepAGT −/− mice at 14 days of WD within overlapped DEGs. (D) Chord graph representing overlapped DEGs corresponding to the top five terms in gene ontology analysis. (E) Normalized read count expression of six target DEGs in hepAGT +/+ and −/− mice after 14 days of WD feeding. False discovery rate-adjusted *p*-values are represented above for each DEG. Abbreviations: AGT: Angiotensinogen; DEG: Differentially expressed gene; FC: Fold change; hepAGT +/+: albumin-Cre 0/0 AGT floxed (f/f) low-density lipoprotein-receptor-deficient background; hepAGT −/−: Albumin-Cre 1/0 AGT floxed (f/f) low-density lipoprotein-receptor-deficient background; WD: Western diet.

To define key molecules mediating the attenuation of WD-induced liver steatosis in hepAGT −/− mice, the two RNA sequencing data were integrated (Figure 4A). We sought to isolate genes that were both increased in association with WD-feeding and altered in hepAGT −/− mice at the initiation phase of steatosis. Since Cluster 4 contained DEGs that were increased in abundance consistent with continued WD-feeding, DEGs in Cluster 4 were compared against all DEGs identified by comparing hepAGT +/+ and −/− mice at 14 days of WD-feeding (Figure 4B). Twenty-four DEGs were overlapped between the two sequencing datasets (Figure 4C). GO enrichment analysis within the overlapped DEGs highlighted six DEGs that were present in the top five biological processes, all of which related to cell division (Figure 4D). These six DEGs represented potential target genes relating to the contribution of AGT to diet-induced steatosis. Among these genes, *Smpd3*, *Mki67*, and *Top2a* demonstrated the highest normalized read count expression (Figure 4E).

### 3.5. mRNA abundance of target cell division genes increased concurrently with steatosis in hepAGT +/+ mice

**Figure 5.**
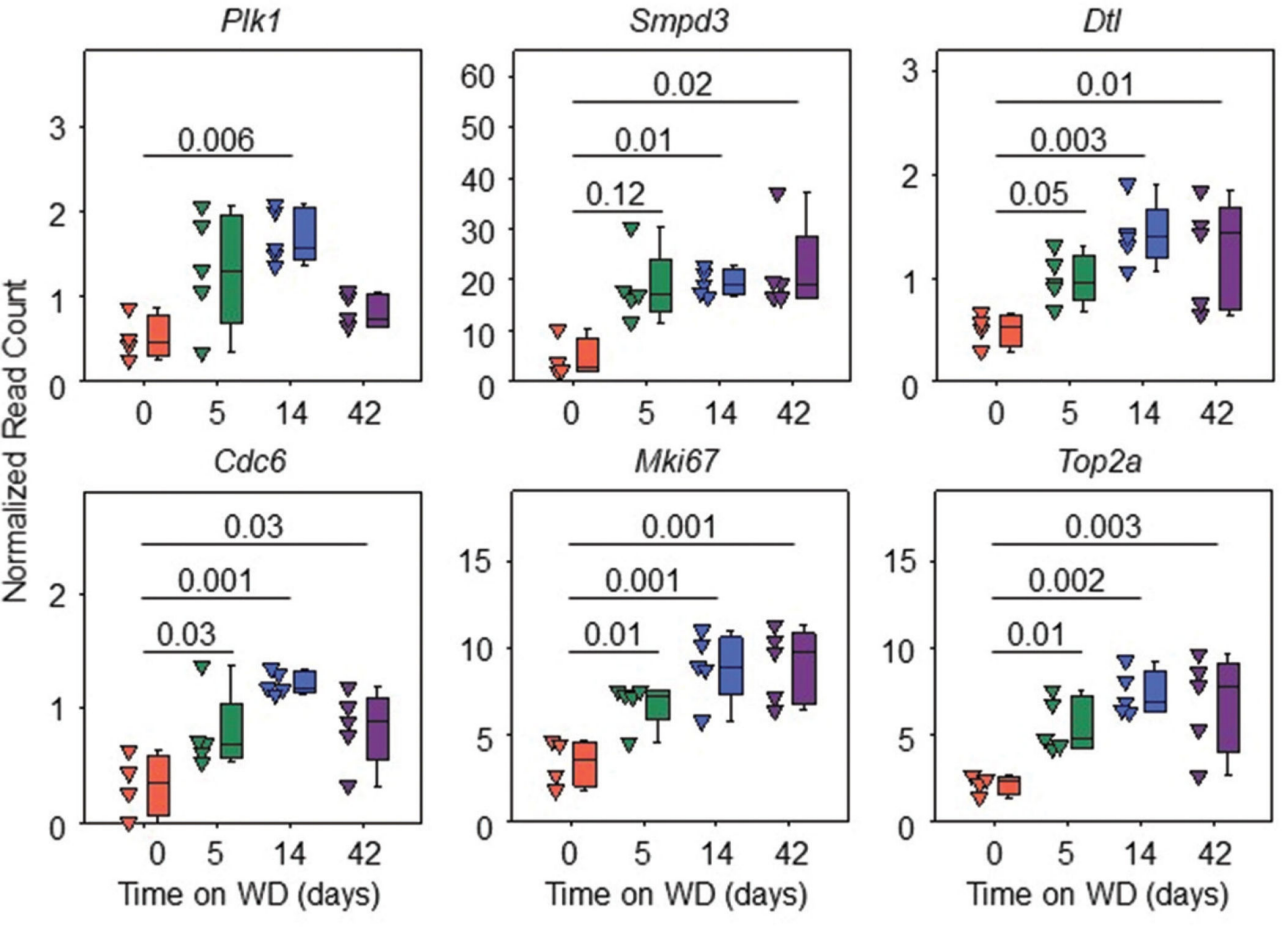
mRNA abundance of target cell division genes increased concurrently with steatosis in hepAGT +/+ mice. Normalized read count expression of target DEGs after 5, 14, and 42 days of WD feeding compared to 0 days in hepAGT +/+ mice. *p-values* compared against 0 days were calculated by Kruskal–Wallis with Dunn’s method for *Plk1* and *Smpd3* or one-way analysis of variance with Holm–Sidak test for *Dtl, Cdc6, Mki67,* and *Top2a. n* = 4 – 5 mice/group. Abbreviations: DEG: Differentially expressed gene; hepAGT +/+: Albumin-Cre 0/0 AGT floxed (f/f) low-density lipoprotein-receptor-deficient background; WD: Western diet.

To verify that the abundance of potential target genes was increased during feeding of WD in hepAGT +/+ mice, normalized read count data for each DEG were analyzed separately. *Plk1* displayed a statistically significant increase in abundance only after 14 days of WD feeding compared to baseline (0 days of WD). Increased abundances of *Smpd3, Dtl, Cdc6, Mki67,* and *Top2a* were detected after 14 and 42 days of feeding WD, which was consistent with the initiation and advanced phases of steatosis in hepAGT +/+ mice (Figure 5).

## 4. Discussion

In the present study, we performed two bulk RNA sequencing analyses to explore the molecular basis underlying the inhibition of WD-induced liver steatosis and protective effects of hepatocyte-specific AGT deletion. We found that genes related to inflammatory biological processes were increased consistently with the progression of steatosis. The mRNA abundance of several key inflammatory cytokines was maximal at an advanced stage of steatosis. Surprisingly, despite the established protective effect against WD-induced steatosis, hepatocyte-specific AGT deficiency altered a select portion of the liver transcriptome, particularly genes related to metabolic and cell division processes, during the initiation phase of steatosis. By integrating data from the two RNA sequencing analyses, we identified five molecules related to cell division: *Smpd3*, *Dtl*, *Cdc6*, *Mki67*, and *Top2a*, as key mediators in suppressing WD-induced steatosis in hepAGT −/− mice.

In this study, liver steatosis was defined histologically by the presence of intrahepatocellular lipid droplet accumulation. Macrovesicular steatosis, a defining feature of metabolic dysfunction-associated fatty liver disease (MAFLD),^29–33^ was observed after 14 days of WD feeding, but not after 5 days, suggesting that 14 days of feeding represents the initiation phase of pathology. This finding is consistent with a previous study reporting hepatic triglyceride content increased in mice after 14 days of a high-fat diet.^34^ In the present study, progressive macrovesicular steatosis was noted after 42 days of WD feeding, consistent with previous studies reporting the presence of macrovesicular steatosis in mice after 56 days of WD or high-fat diet feeding.^28,34^ These observations suggest that 14 days of WD feeding is an optimal interval for investigating the mechanisms underlying liver steatosis that relate to the initiation of this disease.

The primary aim of this study was to identify previously unknown molecules that may interact with AGT during the initiation phase of steatosis. The combination of a time-course RNA sequencing analysis in WD-fed hepAGT +/+ mice and another sequencing analysis comparing hepatocyte-specific AGT genotypes at the initiation phase of steatosis allowed us to isolate target genes with potential roles in both interaction with AGT and development of steatosis. We identified 128 DEGs between hepAGT +/+ and −/− mice at the initiation of steatosis. The integration of the two sequencing data highlighted 24 DEGs. Despite the small number of DEGs, by focusing on the initiation phase, we gain insights into the regulation of potentially causative genes with less interference from genes altered as a consequence of steatosis.

The present study identified five genes (*Smpd3*, *Dtl*, *Cdc6*, *Top2a*, and *Mki67*) as potential mediators in the development of WD-induced liver steatosis. *Smpd3*, encoding neutral sphingomyelinase 2, catalyzes the hydrolysis of the biochemically inert lipid sphingomyelin, leading to an increased abundance of ceramide. Excess ceramide has been implicated in liver inflammation and steatosis, potentially by enhancing endoplasmic reticulum stress, lipid uptake, and triglyceride production in the liver.^35–39^ Both pro- and anti-proliferative functions of ceramide have been reported, explained in part by contextual differences of cell type, subcellular localization, and fatty-acid chain length.^40–43^ The contribution of *Smpd3* and ceramide to liver cell proliferation in the context of WD-induced steatosis remains unclear. However, inhibition of *Smpd3* by genetic or pharmacologic methods attenuates hepatocyte lipid accumulation and pathologic features of MAFLD *in vitro*.^36,39^ *Dtl*, which encodes denticleless E3 ubiquitin protein ligase homolog, plays a key role in the cell cycle as a substrate receptor for cullin-RING ligases and regulates replication licensing, cell cycle control, and chromatin modification-associated proteins.^44,45^ Overexpression of *Dtl* promotes hepatocellular carcinoma (HCC) proliferation and invasion *in vitro* and *in vivo*, while its knockdown attenuates both phenotypes.^46–48^ *Dtl* has been shown to relieve transcriptional repression in HCC cell lines, indicating that *Dtl* may be a causative mediator in liver cell proliferation.^47^ *Cdc6* (encoding cell division cycle 6), *Top2a* (encoding DNA topoisomerase II alpha), and *Mki67* (encoding Ki67) play critical roles in the cell cycle. *Cdc6* functions in the assembly of prereplicative complexes at origins of replication and participates in checkpoint control before mitosis. Increased expression of *Cdc6* may promote DNA hyperreplication or oncogenesis.^49^ Overexpression of *Cdc6* in HCC cells promoted cell proliferation and metastatic markers.^50^ *Top2a* is a crucial part of the DNA replisome and regulates chromatin topology by catalyzing transient DNA double-stranded breaks and facilitating the relief of super-helical stress.^51^ *In vitro* and *in vivo* knockdown and overexpression of *Top2a* can inhibit or promote HCC proliferation and metastasis, respectively.^52^ Ki67 is a well-known marker of cellular proliferation and plays a key role in mitosis by preventing aggregation of mitotic chromosomes.^53–57^ Despite its universal expression in proliferative cells, Ki67 is dispensable for proliferation using several established cancer cell lines, yet its knockdown reduced tumorigenesis potentially through altered chromatin accessibility.^58^ However, *in vitro* studies investigating the role of *Dtl*, *Cdc6*, *Top2a*, and *Mki67* in lipid-treated hepatocytes are needed to investigate their potential causative roles in liver steatosis.

Elevated mRNA abundances of *Dtl*, *Top2a*, *Cdc6*, and *Mki67* are associated with highly proliferative phenotypes in HCC, a disease that can emerge as a long-term consequence of liver steatosis.^48,50,59–63^ We detected increased mRNA abundance of *Dtl*, *Top2a*, *Cdc6*, and *Mki67* in liver before development of macrovesicular steatosis in hepAGT +/+ mice, suggesting that cell proliferation precedes WD-induced liver pathology. This observation is consistent with several recent reports which indicate that early liver proliferation is a key driver of hepatic steatosis.^56,64,65^ Expression of *Top2a*, *Cdc6*, and *Mki67* is dynamically regulated within the cell cycle,^57,66,67^ suggesting that the upregulation of these genes by WD feeding may occur subsequent to proliferative intracellular signaling. *Dtl* relieves transcriptional repression through ubiquitination of several transcriptional modulators.^47,68^ These data suggest that *Dtl* may promote cell proliferation and downstream upregulation of *Top2a*, *Cdc6*, and *Mki67*.^69^ Interestingly, *Smpd3* is often reported as a tumor-suppressing gene in HCC. Overexpression of *Smpd3* in HCC reduced cell proliferation.^70^ *In vitro*, overexpression of *Smpd3* has been shown to promote palmitate-induced insulin resistance.^39^ Disruption of PI3K/Akt signaling and enhanced stress responses resulting from insulin resistance suggest a potential opposing relationship between *Smpd3* and *Dtl* in the context of WD-induced steatosis. While our findings and previous literature suggest potential mechanisms by which *Smpd3*, *Dtl*, *Cdc6*, *Top2a*, and *Mki67* contribute to liver steatosis, further study is needed to clarify their precise roles. Taken together, it is important to understand the complex interplay between WD, AGT, and hepatocyte proliferation in the pathophysiology of liver steatosis.

In the present study, bulk RNA sequencing was performed to determine transcriptomic alterations in livers. A key limitation of bulk RNA sequencing is its inability to resolve cellular heterogeneity, which precludes the identification of cell-specific changes in gene expression.^71^ The responses of heterogenous liver cell populations to WD-feeding, particularly in respect to hepatocyte zonality and the regulatory roles of parenchymal cells, remain critical areas of interest in understanding the link between AGT and liver steatosis. As such, single-cell, single-nuclei, and spatial transcriptomic approaches represent promising tools for future investigations into the relationship between the target genes identified in this study and their potential contributions to AGT-mediated liver steatosis.

The present study used a ketamine/xylazine cocktail to euthanize mice that has the potential to impact metabolic organs, such as the liver.^72^ However, in the present study, mice were rapidly perfused with cold saline by left ventricular puncture, and livers were immediately processed for RNA extraction. Furthermore, all study mice were euthanized and processed in the same manner in a random order. Therefore, we infer that any variation in liver transcriptomes due to the euthanasia method would not interfere with data analyses and interpretation.

## 5. Conclusion

Our integrated transcriptomic analyses identified five target genes related to cell division that may contribute to the development of liver steatosis mediated by WD and AGT. This study provides insights into the role of the RAS in WD-induced hepatic steatosis.

## Data Availability

RNA sequencing data (raw FASTQ and aligned data) are publicly available at the gene expression omnibus (GEO) repository (GSE291082). The data that support the findings of this study are also available from the corresponding authors on reasonable request.
